# Assessing Mitochondrial Function, Oxidative Stress and Vitamin D Status in Dark‐Skinned Patients With Keloid Disease

**DOI:** 10.1155/drp/7086830

**Published:** 2026-10-04

**Authors:** Grant Hattingh, Janette Bester, Jaco Kotze, Alisa Phulukdaree, Evangeline Nortje

**Affiliations:** ^1^ Department of Physiology School of Medicine Faculty of Health Sciences University of Pretoria Pretoria South Africa up.ac.za

**Keywords:** keloid, mitochondria, oxidative stress, vitamin D, wound healing

## Abstract

**Background:**

Keloid disease, marked by excessive fibrous tissue growth, is prevalent in dark‐skinned individuals. Mitochondrial integrity is essential for wound healing through cytoskeletal remodelling. Altered mitochondrial morphology and reactive oxygen species (ROS) signalling may underlie keloid pathology, which may be worsened by vitamin D insufficiency.

**Aim:**

To investigate vitamin D status, mitochondrial function and oxidative stress in keloid disease.

**Methods:**

Keloid tissue and whole blood were collected from 44 dark‐skinned patients (18–65 years). Plasma vitamin D levels were quantified by routine analysis. Plasma malondialdehyde (MDA), a lipid peroxidation marker, was measured using the thiobarbituric acid reactive substance assay. Mitochondrial DNA (mtDNA) integrity was assessed by quantitative PCR in keloid tissue, and mitochondrial morphology was examined with transmission electron microscopy (TEM).

**Results:**

Among the 44 patients, 30 had normal vitamin D levels, 13 had low levels and 1 was deficient. Oxidative stress marker, MDA and mtDNA integrity were similar across vitamin D groups. No association was observed between vitamin D and plasma MDA levels. An inverse relationship emerged between mtDNA integrity and plasma MDA (*r* = −0.5225, CI −0.751–−0.0182, *p* = 0.0036), while vitamin D positively correlated with mtDNA integrity (*r* = 0.402, CI 0.052–0.665, *p* = 0.0224). TEM revealed subtle mitochondrial alterations in patients with low vitamin D, including reduced size, and double‐membrane outlines were visible in several micrographs, whereas others showed less pronounced boundary contrast.

**Conclusion:**

Vitamin D insufficiency was uncommon in this cohort. While oxidative stress marker, MDA and mtDNA integrity did not differ by vitamin D status, structural mitochondrial changes were observed in dark‐skinned keloid patients with low vitamin D.

## 1. Background

Keloid disease is a chronic skin disorder characterised by excessive scar tissue formation following skin injury. Unlike normal scars, keloids extend beyond the boundaries of the original wound, resulting in raised, firm and often painful or itchy lesions [1]. Although benign, keloids can cause considerable functional and psychological distress owing to their persistent nature [1, 2]. The precise mechanisms underlying keloid formation remain unclear, but the condition is believed to be associated with dysregulated wound healing processes, including fibroblast hyperproliferation, excessive collagen deposition and inflammation [3].

Recent research has suggested that mitochondrial function and oxidative stress may play key roles in keloid development. Mitochondrial dysfunction can lead to excessive production of reactive oxygen species (ROS), contributing to oxidative stress, which exacerbates fibroblast activity and inflammation. Vitamin D has been identified as a crucial factor in wound healing, and its deficiency may increase the susceptibility to keloid formation [4–7]. Investigating the relationship between mitochondrial dysfunction, oxidative stress and vitamin D levels may provide insights into novel therapeutic approaches for keloid treatment.

Keloids are dermal tumours resulting from an overactive wound healing response. They typically develop within 3 months of skin injury but may take years to appear. Unlike normal scars, keloids do not regress and often grow indefinitely. Fibroblasts are essential for maintaining and repairing connective tissues by producing extracellular matrix (ECM) components, including collagen and elastin. In keloid formation, fibroblast activity may be dysregulated by hyperproliferation, excessive collagen production, aberrant TGF beta signalling pathways and/or reduced apoptosis, which prevents excessive growth [1, 2, 8, 9].

Chronic inflammation is a key feature of keloids. Inflammatory cells, such as lymphocytes and macrophages, are frequently observed within keloid tissues, indicating that prolonged inflammation contributes to abnormal scar growth and maintenance. The persistent inflammatory response may further stimulate fibroblast activity and collagen synthesis, thereby perpetuating excessive scar formation [10, 11].

Although significant progress has been made in understanding keloid pathophysiology, its primary cause and treatment are still unknown [12]. When surgical excision is the only method used, there is a recurrence rate of 45%–100% for the keloid to start forming again [13]. There are many treatment possibilities available that have been supported, including corticosteroids, 5‐fluorouracil, bleomycin, interferon, cryotherapy and verapamil, that have not yet produced optimal treatment results [14]. Keloids have a high recurrence rate and, therefore, place an additional burden on the South African healthcare system. Understanding the pathophysiology of keloids provides a crucial foundation for exploring the role of mitochondrial function in keloid disease, as mitochondrial integrity is recognised as a key factor that influences keloid development and persistence.

The roles of mitochondrial dysfunction and oxidative stress in keloid formation are an emerging area of research. Mitochondria are essential for cellular energy production, and their dysfunction can lead to increased ROS levels, which promote oxidative stress. Oxidative stress has been implicated in abnormal wound healing as it enhances fibroblast proliferation and inflammatory responses, potentially worsening keloid formation [4, 15–19]. The role of oxidative stress in keloid formation underscores the need for effective antioxidant defence mechanisms to neutralise excess ROS and potentially mitigate the damaging effects that contribute to keloid scar development [7, 20, 21].

Recent clinical studies have further highlighted the relevance of vitamin D in keloid management. Hassan et al. demonstrated that intralesional vitamin D injections reduced keloid and hypertrophic scar size, suggesting a potential therapeutic role [22]. Similarly, Lee et al. reported that lower preoperative vitamin D levels were associated with increased keloid recurrence following surgical excision [23]. These findings support the clinical importance of evaluating vitamin D status in keloid patients and provide additional justification for its inclusion in the present study.

Vitamin D, known for its immunomodulatory and antifibrotic properties, may also play a crucial role in keloid pathogenesis. Vitamin D deficiency has been linked to impaired wound healing and excessive scar formation. Understanding the interplay between vitamin D levels, mitochondrial function and oxidative stress, using plasma malondialdehyde (MDA) as a marker, may offer new therapeutic targets for keloid prevention and treatment. The Fitzpatrick scale is a numerical classification scheme for human skin colour. There are six different types that start from (I) pale white to (VI) dark brown to black skin (deeply pigmented dark brown to black skin). Keloids are usually seen in populations with Fitzpatrick skin types (IV–VI) [24]. Vitamin D deficiency is more prevalent in dark‐skinned populations because of the higher melanin content of the skin [25, 26]. A deficiency in vitamin D within dark‐skinned populations may cause an overactive inflammatory response to skin injury, which can delay the wound healing process, making it more difficult for the body to resolve inflammation and promote proper tissue remodelling [27–29].

Emerging research has indicated that mitochondrial dysfunction and oxidative stress play crucial roles in keloid formation, offering potential targets for novel therapies. Low vitamin D levels, particularly in darker‐skinned individuals, may further contribute to keloid development. Although racial and ethnic disparities in keloid prevalence are well documented, their impact on disease progression is not fully understood. This study aimed to address these research gaps by examining the ultrastructure and interplay between mitochondrial function, oxidative stress and vitamin D levels in dark‐skinned patients with keloid disease.

## 2. Materials and Methods

### 2.1. Study Design and Sample Collection

This study was approved by the Faculty of Health Sciences Research Ethics Committee (Ethics Reference No. 532/2022). Patient recruitment and sample collection were conducted at the Plastic Surgery outpatient clinics of Steve Biko Academic Hospital (SBAH), Kalafong Hospital and Chris Hani Baragwanath Academic Hospital (CHBAH). This was a cross‐sectional, analytical study. A consecutive convenience sampling strategy was employed in which all eligible patients presenting at the participating clinics during the recruitment period were invited to participate in the study. Given the complexity of keloid disease and the limited number of patients who met the specific inclusion criteria, this approach was the most practical means of ensuring adequate participant enrolment. The final sample size was 102 participants, which was calculated based on prior metabolomic studies of fibrotic diseases. This number was determined to provide sufficient statistical power to detect significant differences in immune and metabolic markers between the low and normal vitamin D groups. We were only able to recruit 44 participants due to time and financial limitations. Although an a priori sample size calculation indicated that 102 participants would be required for adequate power, logistical constraints limited recruitment to 44 participants. Therefore, this study is exploratory and hypothesis‐generating. Whole blood was collected in 5‐mL lithium‐heparin tubes by venepuncture (4 mL per sample), and tissue samples (5 mm) were collected following excision in the theatre during routine clinical removal of keloid scars. Patients of any biological sex, dark‐skinned with Fitzpatrick skin type between IV and VI and between 18 and 65 years of age who provided informed consent were included. They were clinically diagnosed with keloid disease. Patients were excluded if they had a history of or currently presented with chronic renal disease or renal failure, chronic liver disease or liver failure, chronic medication, smoking habits, human immunodeficiency virus (HIV) positivity, vitamin supplements or any known immune‐compromised conditions.

### 2.2. Experimental Procedures

#### 2.2.1. Vitamin D Quantification

Vitamin D status was assessed by measuring 25‐hydroxyvitamin D levels using competitive chemiluminescence immunoassay (CLIA). Plasma was obtained from the two Li‐hep blood tubes as described previously and aliquoted into 1.5‐mL plasma tubes. Vitamin D levels were reported in ng/mL, and patients were categorised into two groups based on predefined clinical reference ranges, with 25(OH) *D* < 20 ng/mL classified as low (deficient) and 20–60 ng/mL as a normal vitamin D level [30]. The single participant with vitamin D deficiency (< 20 ng/mL) was excluded from subgroup comparisons because *n* = 1 does not permit valid statistical inference or variance estimation. Including this participant would artificially skew group comparisons; therefore, only normal and insufficient groups were analysed. Vitamin D levels (total hydroxyvitamin *D*_2_ and *D*_3_ 25(OH)D) were determined using the electrochemiluminescence (ECL) 2‐step competitive method (Beckman Coulter DXI). The instrument was used to analyse vitamin D levels by mixing a patient sample with labelled vitamin D molecules that compete for binding to specific antibodies. The light emitted from the ECL reaction is inversely proportional to the vitamin D concentration, with higher vitamin D levels producing lower luminescence. The analysis was conducted following the sample preparation protocols outlined in the laboratory standard operating procedures (SOPs). Internal quality controls were included in each run to ensure analytical accuracy.

#### 2.2.2. Quantitative Polymerase Chain Reaction

Deoxyribonucleic acids were extracted from whole blood using a Quick‐DNA MiniPrep kit. Whole blood was mixed in a 1:4 ratio with the genomic lysis buffer, 100 μL of whole blood and 400 μL of genomic lysis buffer. The mixture was then transferred to a Zymo‐Spin IICR Column in a collection tube and centrifuged at 10,000 × g for 1 min at 4°C. The collection tube was discarded using a flow‐through. The Zymo‐Spin IICR Column was then transferred to a sterile collection tube, and 200 μL of DNA prewash buffer was added to the spin column, which was then centrifuged at 10,000 × g for 1 min at 4°C. Next, 500 μL of gDNA wash buffer was added to the spin column, and the column was centrifuged at 10,000 × g for 1 min at 4°C. The spin column was transferred to a clean microcentrifuge tube, and 50 μL of DNA elution buffer was added. This was then incubated for 15 min at room temperature (RT) and centrifuged at the top speed (18,407 × g) for 30 s at 4°C to elute the DNA. Isolated DNA was quantified using a NanoDrop 2000 spectrophotometer. The spectrophotometer was always calibrated for each use, with a blank using nuclease‐free water. The eluted DNA was stored in a freezer box at −20°C until further analysis.

To ensure the accuracy and reproducibility of qPCR results, several optimisation steps were performed to determine optimal primer and DNA template concentrations and to optimise cycle conditions to maximise amplification efficiency and minimise variability. The final qPCR conditions used were DNA template standardised to 10 ng/μL in a reaction volume of 12.5 μL consisting of 6.25 μL SYBR Green Supermix (BioRad, Hercules, CA, USA), 500 nM of forward (5′‐TGAGGCCAAATATCATTCTGAGGGC‐3′) and reverse primer (5′‐TGCACCTGCTCTCTGTGATTATGACTATCCCACAGTC‐3′). Changes in mitochondrial DNA (mtDNA) amplification were calculated based on the 2^−delta delta cycle threshold^ (2^−ΔΔCt^) method described by Livak and Schmittgen [31] and reported as percentage mtDNA amplification relative to the housekeeping gene *β*‐globin (sense: 5′‐ACATGATTAGCAAAGGGCCTAGCTTGACTCAGA‐3’; antisense: 5′‐TGCACCTGCTCTGTGATTATGATATCCCACAGTC‐3′), which was amplified simultaneously for each sample. The cycling conditions included an initial denaturation step (94°C for 3 min), followed by 38 cycles of denaturation (94°C for 20 s), annealing (58°C for 10 s), extension (72°C for 1 min) and plate reading. This was followed by a melting curve and final hold (25°C for 5 min) using a Roche LightCycler 480 (Roche Diagnostics). *β*‐Globin was validated as the reference gene prior to analysis. Reference gene suitability was confirmed by assessing amplification efficiency (acceptable range 90%–110%), linearity (*R* ^2^ > 0.99) and consistent Cvalues across all samples. *β*‐Globin expression showed no significant variation between vitamin D–normal and vitamin D–insufficient groups, indicating stable expression under the experimental conditions. Melt‐curve analysis confirmed a single, specific amplification product without primer‐dimer formation. These validation steps support the use of *β*‐globin as an appropriate housekeeping gene for normalisation of mtDNA amplification.

The relative mtDNA copy number was calculated, and all samples were run in triplicate. Negative controls were included to ensure no contamination. The results are reported as the relative fold change in mtDNA amplification in the vitamin D–low group compared with patients with normal vitamin D levels. The single sample with vitamin D deficiency was excluded from the analysis.

#### 2.2.3. Thiobarbituric Acid Reactive Substance (TBARS) Assay

Plasma samples were removed from the −80°C freezer and left at RT until thawing. Once the samples were thawed, 250 μL of the plasma sample was added to a glass tube and labelled. Next, 100 μL 2% H_3_PO_4_ was added to the glass tube and vortexed for 3 s. Subsequently, 200 μL of 7% H_3_PO_4_ was added to the glass tube and vortexed for 3 s. Thereafter, 200 μL TBA/BHT was added to the glass tube. A positive control was prepared as follows in a glass tube: 100 μL of 2% H_3_PO_4_, 200 μL of 7% H3PO4, 200 μL of TBA/BHT and 0.5 μL of MDA in 249.5 μL ionised water. A negative control was prepared in a glass tube as follows: 100 μL of 2% H_3_PO_4_, 200 μL of 7% H3PO4, 200 μL of TBA/BHT and 250 μL of 3 mM HCL. When the glass tubes containing the samples were boiled in a beaker, at least one positive and one negative control was included. The assembly was then heated on a hot plate at 100°C for 15 min. The positive control was extracted after 5 min or once it exhibited a pink hue. Subsequently, all the glass tubes were transferred to a tube rack for equilibration at RT. Subsequently, 1 mL of 100% butanol was added to each glass tube containing the samples, and the samples were vortexed for 10 s to facilitate phase separation. The butanol phase was then carefully aspirated, and 200 μL was aliquoted into a 96‐well microtiter plate in triplicate. The absorbance of the MDA‐TBA complex was measured using a spectrophotometer at 532 nm with a reference wavelength of 600 nm, and plasma MDA levels were calculated in nM/mL. All samples were analysed in triplicate to ensure accuracy and reproducibility.

#### 2.2.4. Transmission Electron Microscopy (TEM)

Keloid tissue samples were sectioned into approximately 1 mm^3^ fragments and placed in a 2.5% glutaraldehyde/formaldehyde (GA/FA) fixative solution (composed of 1 mL glutaraldehyde, 1 mL formaldehyde, 3 mL distilled water and 5 mL 0.1M PBS) for 1 h at RT. Following fixation, the tissues were rinsed thrice with PBS, with each wash lasting 15 min. Post‐fixation was performed in a fume hood using 1% osmium tetroxide for 1 h at RT, followed by an additional series of 0.1M PBS rinses.

#### 2.2.5. Dehydration and Embedding

Tissue samples were dehydrated using a graded ethanol series (30%, 50%, 70%, 90% and three changes of 100%) for 15 min at each concentration. Tissue samples were then left overnight in fresh 100% ethanol. After dehydration, the samples were infiltrated for 30 min with a 50:50 mixture of 100% ethanol and a resin prepared for 5 g of tissue from 2.617 g Agar 100, 1.767 g methyl nadic anhydride, 0.683 g dodecenyl succinic anhydride and 0.10 g dimethylaminoethanol.

The prepared resin mixture was then placed in a 1.5 mL test tube with the sample and an identifying label together with 100% ethanol to form a 50:50 mixture and mixed well. The test tube was then slowly rotated at 30 rpm for 30 min. The sections were then placed in a 100% resin mixture and rotated again at 30 rpm for an additional 4 h. The resin mixture and allocated sample numbers were then transferred to moulds (Advanced Laboratory Solutions, SA). The moulds were then placed in an oven at 60°C for 38 h for polymerisation.

#### 2.2.6. Ultramicrotome Sectioning and Staining

The samples were removed from the moulds and trimmed using a single‐edge razor knife. After the optimal cutting area was obtained, the sample was cut with an ultramicrotome (Leica, HistoCore BIOCUT) glass knife until the surface was smooth and the sample was fully cut. The sample was further cut with a DiATOME diamond knife with a thickness of 50–90 nm (until a gold‐like colour started to form), picked up with a copper grid (Advanced Laboratory Solutions, SA) and placed in a grid holder (Advanced Laboratory Solutions, SA). The sample was then placed in an enclosed container to prevent photooxidation and contrasted with uranyl acetate for 5 min at RT with the container closed, followed by 2 min in lead citrate at RT with the container closed.

#### 2.2.7. Imaging and Mitochondrial Analysis

TEM was performed on a JEOL JEM‐2100F microscope at 120 kV. For each patient, at least three micrographs were randomly captured at 5000x, 10,000x and 20,000x magnification to analyse intracellular organelles with a focus on mitochondria. Mitochondrial size, membrane integrity and cell counts were analysed using ImageJ software. Manual thresholding was used to segment mitochondria accurately, and all measurements were averaged across five randomly selected micrographs per sample for statistical analysis. The ultrastructural assessment was qualitative in nature; no blinded scoring system or quantitative morphometric analysis was applied.

#### 2.2.8. Statistical Analysis

Statistical analyses were performed using GraphPad Prism 9.0 (San Diego, CA, USA). All data were tested for normality. Only vitamin D levels passed the normality test; therefore, parametric tests were performed on the data. Nonparametric tests were applied to all the other variables. Data were summarised as means, standard deviations, medians, ranges and 95% confidence intervals (CIs) for continuous variables and as frequency, percentage and 95% CI for categorical (binary/discrete) data. The correlation between vitamin D levels and mitochondrial function (mtDNA integrity, mitochondrial morphology via TEM) and oxidative stress marker, plasma MDA (lipid peroxidation via TBARS assay) was assessed using Spearman’s correlation coefficient. Each patient’s mtDNA copy number (from qPCR results), TBARS‐derived MDA levels and TEM‐determined mitochondrial morphology were compared with their corresponding vitamin D levels to identify potential trends or correlations. For TEM analyses, patient‐level averages were used as independent observations. Individual mitochondria were not treated as independent datapoints. Statistical significance was set at *p* < 0.05.

## 3. Results

Keloid patients with normal vitamin D levels and those with vitamin D insufficiency were evaluated for all analyses, as there were too few values to test (*n* = 1) in the vitamin D–deficient group. The age and sex frequencies were statistically similar (*p* > 0.05) and thus did not skew the analysis of other experimental data. Vitamin D levels, when grouped by status, were significantly different from each other, as expected (*p* < 0.0001, Table 1). The relative fold change in mtDNA levels, as an indicator of mtDNA integrity, was similar in both patient groups (*p* = 0.4873), and the same was true for markers of lipid peroxidation (plasma MDA; *p* = 0.6077; Table 1).

**TABLE 1:** Comparison of demographics, vitamin D level, mitochondrial DNA integrity and malondialdehyde in keloid disease patients with vitamin D sufficiency and insufficiency.

	Normal vitamin D (*n* = 30)	Vitamin D insufficient (*n* = 13)	*p* value
Age (years (range))	34 (19–59)	37 (19–64)	0.717
Gender (male, female)	17, 13	9, 4	0.513
Vitamin D (ng/mL)	29.52 ± 5.60	17.22 ± 2.61^∗^	< 0.0001
Mitochondrial DNA integrity (relative fold change)	0.95 (0.19–1.63)	0.35 (0.07–1.81)	0.4873
Malondialdehyde (nM/L)	0.56 (0.43–0.69)	0.55 (0.40–0.66)	0.6077

*Note:* Unpaired *T*‐test with Welch’s correction. Data for vitamin D are represented as mean and standard deviation, as the normality test was passed. Since the data did not meet normality assumptions, mtDNA integrity and plasma malondialdehyde levels were reported as median and interquartile range.

^∗^ *p* < 0.0001.

Although plasma MDA and mtDNA integrity were similar between patients with normal and insufficient vitamin D levels, a correlation analysis was conducted to determine whether a relationship exists between mtDNA integrity, vitamin D levels and plasma MDA levels in the cohort of keloid patients. Interestingly, a weak but positive relationship between vitamin D and viable mtDNA was observed (Spearman *R*, *r* = 0.402, 95% CI: 0.052 – 0.665, *p* = 0.0224, Figure 1A), and an inverse relationship between mtDNA integrity and plasma MDA levels was found (Spearman *R*, *r* = −0.5225, 95% CI: −0.751–−0.0182, *p* = 0.0036, Figure 1B).

**FIGURE 1:**
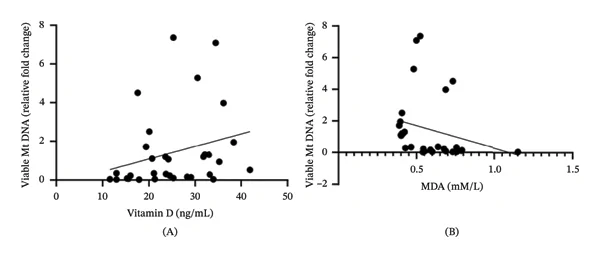
A direct association between mitochondrial DNA integrity and vitamin D levels was found (A). An inverse association between mitochondrial DNA integrity and plasma MDA (malondialdehyde) levels was observed (B).

### 3.1. Analysing Mitochondrial Ultrastructure in Keloid Tissue

The ultrastructure of the keloid tissue, collected and examined under TEM, was analysed, with particular emphasis on the mitochondria. The keloid tissues collected were separated into two patient groups based on vitamin D status: normal (> 20 ng/mL) or low (< 20 ng/mL).

Micrographs of keloid tissue from patients with normal vitamin D levels showed clusters of mitochondria that were present mostly throughout the cell (Figure 2A). In Figure 2B, mitochondria lack a clear contrast of the double membrane structure (red arrows). In Figure 2C, there is one mitochondrion displayed with a clear double‐membrane structure (yellow arrow) and another elongated mitochondrion (red arrow). The mitochondria present in this image exhibit cristae folding within the inner membrane. The mitochondria represented in the yellow block display a bilobed structure, which is rarely seen in this population group. Figure 2D illustrates two bundles of mitochondria (yellow and black). Figure 2E, magnified image of Figure 2D, displays a mitochondrion with a double membrane (red arrow). Figure 2F shows another bundle of mitochondria. Mitochondrial morphology was predominantly round or oval in Figures 2B–D; however, some mitochondria showed a bilobed structure (Figure 2C, yellow block) or an elongated, stretched morphology (Figure 2C, red arrow). The mitochondria are also diminutive, measuring 180–1.5 μm in length.

**FIGURE 2:**
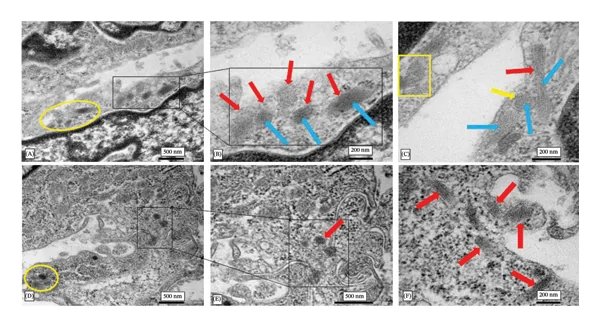
Micrograph of sections through keloid tissue from patients with normal vitamin D levels. (A) illustrates a cluster of mitochondria adjacent to a fibroblast. (B) shows a magnified view of (A) to better visualise mitochondrial morphology. More bundles of mitochondria are seen in (C). (E) provides a close‐up of (D). (E) further details the abundance of mitochondrial clusters in these cells. (A), (D) and (E) were taken at 10,000×, and (B), (C) and (F) were taken at 20,000×. Red arrows point towards the membrane of the mitochondria, whereas the yellow arrow highlights a double membrane layer. Mitochondrial profiles displayed variability in membrane definition, with some showing more distinct double‐membrane boundaries and others appearing less sharply delineated. Cristae visibility also differed across profiles, reflecting morphological diversity.

In Figure 3A, the cell is shown in overview, highlighting a small bundle of mitochondria. Throughout the cell, mitochondria were not overabundant; in this area, they were more numerous than normal. Figure 3B is a close‐up of Figure 3A, showing variable contrast of the membranes around the mitochondria (yellow circles). Figure 3C shows mitochondria displaying visible double membranes, with some exhibiting clearly defined cristae while others show less distinct internal membrane organisation (red arrows). In Figure 3D, a mitochondrion with greater cross‐sectional area and a clearly visible double membrane (red arrow) and well‐defined, properly folded cristae (yellow arrows) is observed. In Figure 3E, two mitochondria with double membranes (red arrows) and adjacent collagen fibrils (yellow arrows) can be seen. In Figure 3F, several mitochondria with double membranes are present (red arrows); the two mitochondria with larger cross‐sectional area also display evidence of properly folded cristae (yellow arrows). Mitochondrial morphology appeared slightly different, as evidenced by the increasingly round or oval shape observed in the images (Figures 3A–F). The size of the mitochondria also increased, as observed in Figures 3A–F, with lengths ranging from 500 nm to 1.5 μm indicating size heterogeneity.

**FIGURE 3:**
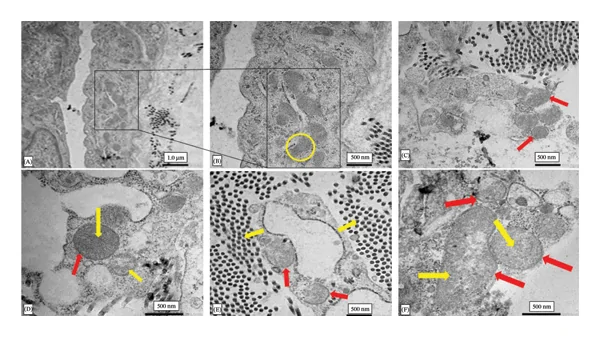
Micrograph of sections through keloid tissue from low vitamin D patients. (A) shows an overview of the cell with a small cluster of mitochondria, taken at 5,000×. (B) demonstrates a magnified image of (A), showing a close‐up of the mitochondria morphology. (C) shows a bundle of mitochondria grouped together. (D) depicts a mitochondrion with greater cross‐sectional area than others in the same field of view. (E) shows two mitochondria organelles next to a vacuole with higher dimensional variability alongside collagen fibrils (yellow arrows). (F) displays mitochondria that exhibit normal morphology. (B)–(F) were taken at 10,000×. Red arrows represent double‐membrane mitochondria. Mitochondria in this group similarly exhibited variation in membrane clarity and cristae prominence. Some profiles showed more pronounced membrane boundaries, while others appeared more diffuse, consistent with the heterogeneity observed across all samples.

## 4. Discussion

This study investigated the mitochondrial ultrastructure, mtDNA integrity, oxidative stress and vitamin D status in dark‐skinned patients with keloid disease. These findings suggest a potential association between mitochondrial dysfunction, increased oxidative stress marker, plasma MDA and vitamin D levels in this population.

A significant difference in vitamin D levels was observed between the normal and low vitamin D groups, consistent with the literature, indicating lower vitamin D levels in dark‐skinned individuals [32, 33]. However, unexpectedly high vitamin D levels were observed in some patients, challenging the assumption that dark‐skinned individuals universally exhibit lower vitamin D levels [32]. Possible explanations include increased sun exposure, dietary intake, genetic factors affecting vitamin D metabolism and the inflammatory state associated with keloid disease [34].

Although dark‐skinned individuals typically exhibit lower vitamin D levels due to increased melanin content, several participants in our cohort presented with unexpectedly high vitamin D levels. This may reflect increased sun exposure, dietary differences or genetic variation in vitamin D metabolism. These contextual factors may explain why vitamin D insufficiency was less prevalent than expected.

Previous studies have linked vitamin D deficiency to keloid formation [35], yet the presence of keloids despite normal vitamin D levels in this cohort suggests that additional factors, such as vitamin D receptor activity, keratinocyte function and metabolic pathways, may influence keloid pathogenesis. Furthermore, these findings highlight potential geographic and contextual differences in vitamin D metabolism among dark‐skinned populations.

The clinical benefits of vitamin D reported in previous studies may relate to its regulatory effects on mitochondrial stability and cellular redox balance. Although our cohort did not show differences in systemic oxidative stress markers between vitamin D groups, the subtle mitochondrial structural changes observed in vitamin D–insufficient patients may reflect early or localized effects not captured by plasma MDA levels. This suggests that vitamin D may influence mitochondrial morphology independently of systemic oxidative stress marker, plasma MDA, aligning with clinical findings that vitamin D improves scar outcomes even when biochemical markers remain unchanged.

In addition, Figure 1 presents the results of the correlation analyses assessing the relationships between vitamin D levels, viable mtDNA integrity and plasma MDA levels in keloid patients. Given the established roles of oxidative stress and mitochondrial dysfunction in pathological fibrosis, these analyses aimed to elucidate the potential interactions between these factors in keloid diseases. Age did not differ significantly between vitamin D groups (*p* = 0.717), reducing the likelihood of age‐related confounding.

Figure 1A suggests that higher systemic vitamin D levels are associated with enhanced mitochondrial integrity. Vitamin D has been implicated in modulating oxidative stress and mitochondrial homeostasis by regulating antioxidant enzymes and inhibiting ROS generation. The observed relationship, albeit modest, aligns with previous findings and suggests a role for vitamin D in mitochondrial protection [36]. However, the considerable variability in mtDNA integrity at comparable vitamin D concentrations implies that additional biological factors, such as genetic predisposition, mitochondrial biogenesis efficiency and differential cellular responses to oxidative stress, may influence this association.

Conversely, as shown in Figure 1B, the observed negative correlation suggests that elevated oxidative stress marker, plasma MDA, compromises mtDNA integrity, potentially through oxidative modifications of mitochondrial lipids, proteins and nucleic acids. This aligns with the existing literature, indicating that increased ROS generation and lipid peroxidation contribute to mitochondrial dysfunction, which, in turn, may exacerbate the aberrant fibroproliferative response observed in keloid pathogenesis [37, 38].

These findings provide valuable insights into the interplay between the vitamin D status, oxidative stress marker, plasma MDA and mitochondrial function in keloid disease. While these data indicate that vitamin Ds correlation analysis suggests a potential association with mitochondrial integrity, oxidative stress appears to exert a deleterious impact on mtDNA stability. Given that mitochondrial dysfunction is a hallmark of chronic fibrosis (37) and aberrant wound healing [39], these results underscore the need for further mechanistic studies to elucidate the role of vitamin D supplementation in oxidative stress‐induced mitochondrial impairment in keloid patients.

Furthermore, TEM analysis revealed mitochondrial changes in both groups, including altered mitochondrial size and membrane integrity. Elevated ROS levels in keloid cells may contribute to mitochondrial damage and reduced mitochondrial abundance [38], reinforcing the link between oxidative stress and mitochondrial dysfunction in keloid pathology.

Patients with normal vitamin D tended to show more typical mitochondrial morphology; although this association does not imply a protective effect, some patients with normal vitamin D still exhibited mitochondrial abnormalities, including altered double membranes and organelle morphologies. This suggests that while vitamin D may mitigate mitochondrial damage, it is not the sole determinant of mitochondrial integrity in keloid disease [40]. Additional factors, such as genetic predisposition, diet, other markers of oxidative stress and inflammation, likely contribute to mitochondrial dysfunction in keloid pathology [41]. Oxidative stress was assessed using a single marker (plasma MDA), which reflects only systemic lipid peroxidation. Comprehensive oxidative stress profiling typically requires multiple biomarkers [42]. Body mass index and obesity status were not available for all participants and could not be controlled for. Age differences were assessed and found not to differ significantly between groups (*p* = 0.717), although age‐related oxidative stress variation cannot be fully excluded. These findings underscore the complexity of the role of vitamin D in keloid progression and highlight the need for further research to explore potential therapeutic strategies targeting antioxidants and mitochondrial health in keloid patients.

## 5. Conclusion

In conclusion, this study provides preliminary evidence supporting an association between mitochondrial alterations, oxidative stress and vitamin D status in dark‐skinned patients with keloid disease. These findings underscore the critical role of mitochondrial health in keloid pathogenesis and suggest that mitochondrial dysfunction may be a central driver of keloid formation. Although vitamin D appears to exert a minor protective effect, its influence is nuanced and warrants further investigation.

### 5.1. Study Limitations

Although this study produced valuable findings, several considerations could enhance future research. First, owing to the limited number of data points in the low vitamin D group, categorical pairwise analysis with this group was not possible. However, this prompted the incorporation of continuous variable analysis across the range of vitamin D concentrations, providing a broader view of the relationship between vitamin D levels and correlated variables. Therefore, this approach leveraged the full dataset to detect trends in correlation patterns while ensuring statistically valid comparisons between variables. The absence of an adequately powered vitamin D deficient subgroup limited interpretation of deficiency‐specific effects. Second, although TEM analysis provides important insights into mitochondrial morphology, incorporating functional assays in future work could further elucidate mitochondrial bioenergetic capacity, complementing morphological observations. ATP BMI and obesity status were not recorded and therefore could not be controlled for, despite their known influence on plasma MDA levels. Clinical characteristics such as keloid duration, recurrence history, anatomical location and prior treatments were not consistently available and could not be included. Lifestyle factors influencing vitamin D status, including sunlight exposure and dietary intake, were not assessed.

## Author Contributions

Grant Hattingh is the primary student who obtained informed consent, performed sample preparation, conducted experiments and wrote the first draft of the manuscript. Alisa Phulukdaree conceptualised the project and trained Grant Hattingh on molecular techniques and sample preparation, completed the data analysis and statistics and edited and formatted the final draft of the paper. Evangeline Nortje and Janette Bester supervised Grant Hattingh and provided training on microscopy, micrograph interpretation and writing the first draft of the manuscript and final editing of the manuscript. Jaco Kotze contributed to conceptualisation of the vitamin D and keloid patient relationship aspect of the project and assisted with patient recruitment, informed consent and sample collection.

## Funding

Funding for the project was received from Prof. Janette Bester’s NRF Incentive funding (RA201211579249).

## Disclosure

This work has been submitted in a more comprehensive form in the format of a thesis for degree purposes [40].

## Ethics Statement

This study was approved by the Faculty of Health Sciences Research Ethics Committee (Ethics Reference No. 532/2022). Patient recruitment and sample collection were conducted at the Plastic Surgery outpatient clinics of Steve Biko Academic Hospital (SBAH), Kalafong Hospital, and Chris Hani Baragwanath Academic Hospital (CHBAH). This was a cross‐sectional, analytical study.

## Consent

Informed consent was obtained prior to sample collection.

## Conflicts of Interest

The authors declare no conflicts of interest.

## Data Availability

The data that support the findings of this study are available from the corresponding author upon reasonable request.
